# Selection of optimal quantile protein biomarkers based on cell-level immunohistochemistry data

**DOI:** 10.1186/s12859-023-05408-8

**Published:** 2023-07-22

**Authors:** Misung Yi, Tingting Zhan, Amy R. Peck, Jeffrey A. Hooke, Albert J. Kovatich, Craig D. Shriver, Hai Hu, Yunguang Sun, Hallgeir Rui, Inna Chervoneva

**Affiliations:** 1 Division of Biostatistics, Department of Pharmacology and Experimental Therapeutics, Sidney Kimmel Medical College, Thomas Jefferson University, Philadelphia, PA 19107 USA; 2 Department of Pathology, Medical College of Wisconsin, Milwaukee, WI 53226 USA; 3 John P. Murtha Cancer Center, Uniformed Services University and Walter Reed National Military Medical Center, Bethesda, MD USA; 4 Chan Soon-Shiong Institute of Molecular Medicine at Windber, Windber, PA USA

**Keywords:** Cellular protein expression, Distribution quantiles, Cancer biomarkers, Tissue microarrays, Breast cancer

## Abstract

**Background:**

Protein biomarkers of cancer progression and response to therapy are increasingly important for improving personalized medicine. Advanced quantitative pathology platforms enable measurement of protein expression in tissues at the single-cell level. However, this rich quantitative cell-by-cell biomarker information is most often not exploited. Instead, it is reduced to a single mean across the cells of interest or converted into a simple proportion of binary biomarker-positive or -negative cells.

**Results:**

We investigated the utility of retaining all quantitative information at the single-cell level by considering the values of the quantile function (inverse of the cumulative distribution function) estimated from a sample of cell signal intensity levels in a tumor tissue. An algorithm was developed for selecting optimal cutoffs for dichotomizing cell signal intensity distribution quantiles as predictors of continuous, categorical or survival outcomes. The proposed algorithm was used to select optimal quantile biomarkers of breast cancer progression based on cancer cells’ cell signal intensity levels of nuclear protein Ki-67, Proliferating cell nuclear antigen, Programmed cell death 1 ligand 2, and Progesterone receptor. The performance of the resulting optimal quantile biomarkers was validated and compared to the standard cancer compartment mean signal intensity markers using an independent external validation cohort. For Ki-67, the optimal quantile biomarker was also compared to established biomarkers based on percentages of Ki67-positive cells. For proteins significantly associated with PFS in the external validation cohort, the optimal quantile biomarkers yielded either larger or similar effect size (hazard ratio for progression-free survival) as compared to cancer compartment mean signal intensity biomarkers.

**Conclusion:**

The optimal quantile protein biomarkers yield generally improved prognostic value as compared to the standard protein expression markers. The proposed methodology has a broad application to single-cell data from genomics, transcriptomics, proteomics, or metabolomics studies at the single cell level.

**Supplementary Information:**

The online version contains supplementary material available at 10.1186/s12859-023-05408-8.

## Background

Protein markers in histological sections of malignant tumors hold great promise as predictors of response to therapy and prognosis [1]. Immunohistochemistry (IHC) is the standard way of assessing protein levels in tumor tissues. Historically, pathologists have visually estimated tumor marker levels in qualitative or at best semi-quantitative manner, but improvements in hardware, software and computational capacities are facilitating more objective digital marker quantification [2, 3]. Protein biomarkers of cancer progression and response to therapy are increasingly important for improving personalized medicine. Advanced quantitative pathology platforms enable measurement of protein expression in tissue sections at the single-cell level (cell signal intensity; CSI). However, much of the rich quantitative information of cell-by-cell biomarker expression levels is rarely exploited. For instance, the quantitative marker levels in individual cancer cells of a tumor section are used to dichotomize cells into either negative or positive for the marker, and the tumor is described in terms of percent positive cells [4, 5]. Not only does this method forego important quantitative marker information, but the method suffers from subjective and operator-dependent thresholding into biomarker-positive or -negative cells. An alternative method that does retain quantitative marker information involves reducing individual cell signal intensities to a single mean across the cells of interest (cancer compartment Mean Signal Intensity; ccMSI) [6]. However, it is well known that there is important intra-tumoral cell-to-cell heterogeneity in levels of proteins or protein modifications [7]. This heterogeneity in CSI of protein marker levels is not captured if only the ccMSI is considered. The data that motivates this work include multiple tumor protein markers quantified by fluorescence-based immunohistochemistry (IF-IHC) [8] in two distinct cohorts of patients with invasive breast cancer. The tumor samples were assembled into standard core-based tissue microarrays (TMAs) [9] and IF-IHC was performed for breast cancer relevant proteins including nuclear protein Ki-67 [9, 10], Programmed cell death 1 ligand 2(PD-L2) [11], and Progesterone receptor (PR) [12]. Ki-67 and Proliferating cell nuclear antigen (PCNA) are nuclear proteins associated with cellular proliferation, and Ki-67 is an established surrogate marker of tumor progression. PR is an estrogen receptor stimulated gene and mediates effects of progesterone in breast tissue. PD-L2 is a ligand for Programmed cell death protein-1 (PD-1), an immune checkpoint protein expressed on lymphocytes. PD-L2 is considered as one protein marker in this study based on our successful validation of the PD-L2 antibody used on the IHC assay [13].

**Fig. 1:**
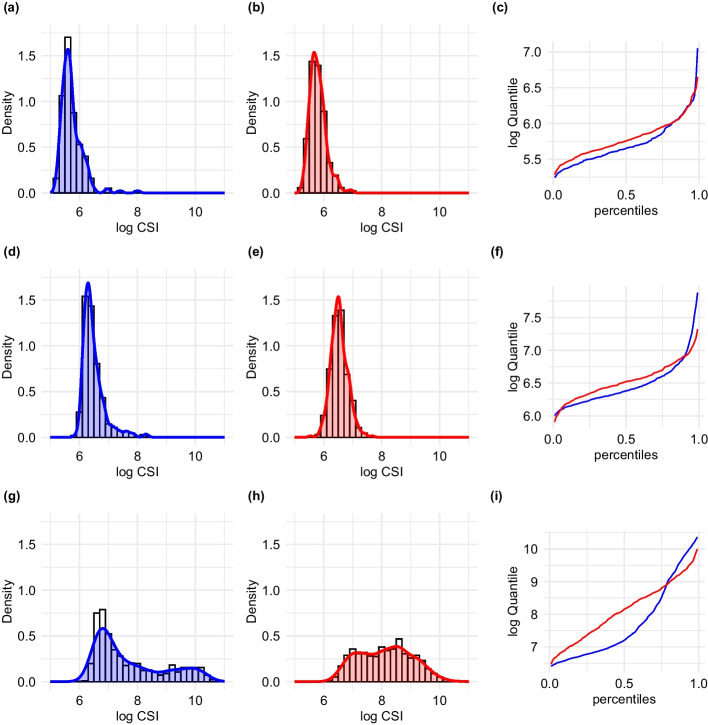
Density and quantile function of CSI for Ki-67 **a** Low ccMSI: 339.94, **b** Low ccMSI: 343.96, **c** Corresponding quantiles functions to the two patients with low ccMSIs in **a** and **b**, **d** Medium ccMSI: 717.41, **e** Medium ccMSI: 719.86, **f** Corresponding quantiles functions to the two patients with medium ccMSIs in **d** and **e**, **g** High ccMSI: 5176.70, **h** High ccMSI: 5040.00, **i** Corresponding quantiles functions to the two patients with high ccMSIs in **g** and **h**

For the purpose of illustration, Fig. 1 shows CSI protein expression distributions of Ki-67 protein in cancer cells in three pairs of breast cancer specimens that pairwise have very similar ccMSI but greatly different CSI distributions. Notably, the differences in CSI distributions and ccMSI are most pronounced for tissues with high ccMSI levels, which tend to be most informative for many proteins. In paired tumors that yield very similar standard ccMSI signal, there can be substantial variability of CSI distributions, including different spread and tails, especially high end tails. Thus, there is a potential to investigate all parts of the CSI distributions as potential predictors of clinical outcomes to improve the prognostic value in comparison to the standard ccMSI.

In this work, we develop a novel approach for deriving tumor biomarkers based on values of empirical quantile function (or simply quantiles) of CSI distributions of protein signals. The empirical quantile function is the inverse of the empirical distribution function [14]. The quantiles are often modeled as responses in quantile regression models [15], meanwhile the use of distribution quantiles as predictors in statistical models has been limited to time series analysis [16, 17]. We propose a selection algorithm that compares dichotomized candidate CSI distribution quantiles to identify the optimal predictor of the continuous, categorical or survival outcome. In the screening cohort, we use repeated split sampling to determine the optimal dichotomization cutoff for each quantile of interest in the training split part and compute the corresponding effect size in the test split part. The effect sizes in test splits are computed for 100-200 splits, and the optimal quantile is selected with the highest median effect size. Validation using an independent external cohort is recommended to confirm the selection of optimal quantiles. In the absence of external data available for validation, internal validation of the data-driven dichotomization by applying the bootstrap-based optimism correction procedure is proposed [18, 19].

The new approach was applied to identify optimal CSI quantile biomarkers based on protein expression of Ki-67, PCNA, PD-L2, and PR in breast cancer tissue. The performance of the resulting optimal quantile biomarkers was validated and compared to the standard ccMSI markers using an independent external validation cohort. For Ki-67, it was also compared to International Ki67 in Breast Cancer Working Group (IKWG) scoring methods by defining high vs. low scoring at commonly used cutoffs of 5%, 15%, 20%, and 30% [20]. For proteins associated with progression-free survival (PFS), the optimal quantile biomarkers yielded either larger (for Ki-67 and PD-L2) or similar (for PR) effect size (hazard ratio for PFS) as compared to ccMSI biomarkers. For PCNA, neither ccMSI nor the optimal quantile biomarker were significant predictors of PFS in the external validation cohort. The proposed algorithm for selecting optimal biomarkers can be also used with any biomedical data that include multiple measures of a candidate predictor per each subject.

## Methods

### Study cohorts

The immunohistochemistry image data were available for a large cohort of breast cancer specimens in a standard core-based tissue microarrays (TMAs). The breast cancer specimens were unselected consecutive cases of primary invasive breast cancer from Thomas Jefferson University Hospital, Philadelphia, PA (screening cohort) and an independent validation cohort from the Clinical Breast Care Program (CBCP) at the Walter Reed National Military Medical Center, Bethesda, MD.

We defined progression event only if it was a documented recurrence or death related to disease. Patients who died from other or unknown causes were censored at the time of death. The screening cohort included 845 non-metastatic hormone positive (HR+, defined as estrogen receptor(ER)+ or progesterone receptor(PR)+) breast cancer patients with 142 progressions and the clinical follow-up ranging from 2 months to 238 months with median follow-up time of 115 months. The external validation cohort included 340 non-metastatic HR+ breast cancer patients with 42 progression events and clinical follow-up ranging from 0.8 months to 297.5 months with a median follow-up time of 148 months. Table 1 summarizes clinico-pathological characteristics of the screening and validation cohorts.

Both screening and validation cohorts included all patients for whom appropriate cell-level immunohistochemistry data were available for at least one, but not necessarily all considered proteins. The cell-level immunohistochemistry data were considered appropriate if the IF-IHC image passed quality control and at least 20 cancer cells were identified in the tissue core. Respectively, the actual sample size for analysis of each protein was lower than 845, as detailed in Additional file 1: Table S1. Similarly, the actual sample size for analysis of each protein in the validation cohort was lower than 340, as detailed in Additional file 1: Table S2.

### Immunohistochemistry and image analysis

Immunostaining was performed on an autostainer (Dako Link). The following antibodies were used for Cy5-tyramide-based fluorescence immunohistochemistry: Ki-67 (Cat#: M7240, Agilent, Santa Clara, CA), PCNA (Cat#: ab29, Abcam, Cambridge, MA), PR (Cat#: M3568, Agilent) and PD-L2 (Cat#: SAB3500395, Sigma-Aldrich, St. Louis, MO). Stained slides were scanned at 20x magnification on the Scanscope laser scanner (Leica/Aperio), and fluorescent images were captured in three channels (DAPI (cell nuclei), Alexa555 (pan-cytokeratin) and Cy5 (protein of interest). Protein signals were quantified from digitized immunohistochemistry image data using the Definiens Tissue Studio software platform [13, 21, 22]. Each image underwent visual inspection and quality control to select appropriate tissue regions and eliminate problem spots. The cell segmentation was performed using the Definiens operator-guided machine learning algorithm supported by cytokeratin staining of carcinoma cells and DAPI staining of cell nuclei. The signal intensities at the pixel level were used to compute the cell signal intensity (CSI) within individual cancer cells at whole cell level (PD-L2) or cell nucleus level (Ki-67, PCNA, PR).

### Optimal quantile selection algorithm

For a sample $\{x_{i},1\le i \le n\}$ of CSI expressions and probability *p*, $0<p<1$, the empirical quantile function $Q_{n}(p)$ is defined as the $k^{th}$ order statistic of the sample, where *k* is such that $(k-1)/n<p<k/n$. For $p=0.01,\dots , 0.99$ and $k=100\times p$, $Q_{n}(p)$ is also known as kth percentile. The empirical quantile function $Q_{n}(p)$ is an estimate of the theoretical (true) quantile function *Q*(*p*), which is the inverse of the distribution function *F*(*x*), that is $Q(F(x))=x$. The well-known median is the 50th percentile, $Q_{n}(0.5)$. It is often used as a robust estimate of location alternative to the mean. The difference in upper tails of CSI distributions translates into differences in *Q*(*p*) values for $p>0.8$, as illustrated in Fig. 1f.

The following algorithm is proposed to identify the optimal *Q*(*p*) predictor of a binary or survival outcome in a screening data set:

- Select the set of quantiles to be evaluated as predictors and the desired ratio for training/test sets.
- Split the data into groups with and without event (e.g. separate patients with and without recurrence).
- Split the group of subjects with event into a training set and a test set randomly with desired ratio. Similarly, split the group of subjects without event into a training set and a test set randomly with desired ratio. Combine training sets with and without event and test sets with and without event.
- For each training/test set pair and each considered quantile,
- Determine the optimal cutoff (e.g. using the R package rpart [23]) in the combined training set.
- Apply the optimal cutoff to the combined test set and estimate the effect size (odds or hazard ratio).
- Repeat steps 3-4 for 100 training/test splits, compute the median log effect size (log odds ratio(OR) or log hazard ratio(HR)) for each quantile
- Rank the absolute values of the median log effect sizes for all considered quantiles and select the optimal quantile with the highest effect size.
- Perform bootstrap-based optimism correction for the selected optimal quantile(s).

In this work, for each random split, 80% of subjects were assigned to the training set and 20% of subjects were assigned to the test set. We considered every fifth quantile starting from the 5th quantile to the 95th quantile plus 99th quantile as candidate predictors of PFS (a total of 20 quantile predictors). Also, we identified quantiles with the second and third highest effect sizes to compare them to the optimal ones.

### Bootstrap-based optimism correction for dichotomizing quantiles

The bootstrap optimism correction procedure [18] is performed as described for a general model selection [19, 24–26]. First, 200-500 bootstrap samples are drawn with replacement from the main sample. In each bootstrap sample, the tree model is used to establish an objective data-driven optimal cutpoint for an optimal quantile. The cutpoint from the current bootstrap sample is used to compute the log OR/HR for dichotomized quantile predictor in the current bootstrap sample (“Bootstrap performance”) and in the main sample (“Test performance”), and the optimism in log OR/HR estimation is computed as the difference between log OR/HR for “Bootstrap performance” and for “Test performance”. The median optimism estimate is computed as the median of optimism estimates over all bootstrap samples. The cutpoint for dichotomizing each selected optimal quantile is also established in the main sample and its “apparent performance” is computed as the log OR/HR for dichotomized quantile in the univariable logistic regression or Cox models. Finally, the optimism-corrected performance estimate is computed by subtracting the median optimism estimate from the apparent performance estimate.

### Multivariable analysis of progression-free survival

For multivariable analysis of progression-free survival (PFS) in the screening cohort of breast cancer patients, multiple imputations were used due to the missing values for clinico-pathologic covariates for some patients (Additional file 1: Table 1). Forty (40) imputed datasets were created using the multivariable Imputation by Chained Equations (MICE) algorithm [27]. For each covariate, missing values were imputed by univariable models for corresponding outcome type using the Fully Conditional Specification [28, 29]. The bootstrap optimism correction algorithm was applied to each imputed data set. Then results for all imputed data sets were averaged using Rubin’s rule [30]. In addition to biomarkers, the multivariable Cox proportional hazards model included the standard clinico-pathological prognostic predictors of PFS: tumor size (< 2 cm, 2-5 cm, or > 5 cm), node status positivity, age at diagnosis, race (White vs. non-White), radiation therapy, chemotherapy, and hormone therapy compliance (the reference category included patients who had hormone therapy and patients with hormone therapy not indicated). The histological grade was included as a strata due to the violation of the proportional hazards assumption.

### External validation of optimal quantile biomarkers

External validation is required for development of new biomarkers. Our quantile selection algorithm includes the internal validation step for the dichotomization cutoff, but performance of the optimal quantile selection has to be validated using an independent data set. The optimal quantile selected for each protein of interest, was evaluated in the external validation cohort as dichotomized into high vs. low categories using the cutoffs identified in the screening cohort. Two types of cutoffs for dichotomizing were considered: (i) the apparent performance cutoff in the entire screening data set; (ii) the median of the cutoffs for 100 random training set samples drawn from the screening data set. The prognostic value of dichotomized optimal quantile biomarkers using both types of cutoffs was evaluated in the univariable and multivariable Cox proportional hazards model. Due to the limited number of progression events in the validation cohort, the initial parsimonious multivariable Cox model was developed (reduced to significant predictors at the level 0.05) without quantile biomarkers and then each quantile biomarker was added to the model separately. The parsimonious multivariable Cox proportional hazards models were used with clinico-pathological prognostic predictors listed in Table 1.

### Dichotomized ccMSI and percentages of Ki-67 positive cells in the validation cohort

We have included for comparison the results of using dichotomized ccMSI of the considered proteins as a predictor of PFS. The apparent performance cutoff was computed in the entire screening data set and applied to dichotomize ccMSI in the external validation cohort. We have also evaluated performance of the standard Ki-67 biomarkers based on dichotomizing proportions of Ki-67 positive cells. The validation cohort included the proportions of Ki-67 positive cells, as evaluated by the pathologist. Previously established cutoffs 5%, 15%, 20%, and 30% for percentages of Ki-67 positive cells [20] were used. Seven patients had multiple tissue cores with different percentages of Ki-67 positive cells, and averaged percentages were used for these patients. All dichotomized ccMSI and Ki-67 positivity biomarkers were evaluated in the univariable and multivariable Cox model the same way as for the optimal quantile biomarkers.

## Results

**Table 1:** Patient and tumor characteristics in screening and external cohort

	Screening cohort (N=845)	External cohort (N=340)
*Recurrence*		
Recurred	142 (16.8%)	42 (12.4%)
Not Recurred	703 (83.2%)	298 (87.6%)
Age	59.9 (13.3)	59.6 (13.1)
*Race*		
White	726 (85.9%)	261 (76.8%)
Non-white	119 (14.1%)	79 (23.2%)
*Histological Grade*		
1	295 (34.9%)	138 (40.6%)
2	362 (42.8%)	141 (41.5%)
3/4	184 (21.8%)	60 (17.6%)
Missing	4 (0.47%)	1 (0.29%)
*Tumor size*		
Tumor Size < 2 cm	541 (64.0%)	218 (64.1%)
Tumor Size 2–5 cm	232 (27.5%)	101 (29.7%)
Tumor Size > 5 cm	72 (8.52%)	21 (6.18%)
*Node*		
Positive	297 (35.1%)	114 (33.5%)
Negative	544 (64.4%)	215 (63.2%)
Unknown	4 (0.47%)	11 (3.24%)
*Her2*		
Positive	82 (9.70%)	27 (7.94%)
Negative	673 (79.6%)	312 (91.8%)
Missing	90 (10.7%)	1 (0.29%)
*Chemotherapy*		
Chemotherapy: Yes	225 (26.6%)	–
Chemotherapy: No	596 (70.5%)	–
Unknown	24 (2.84%)	–
*Radiation*		
Radiation: Yes	334 (39.5%)	–
Radiation: No	489 (57.9%)	–
Unknown	22 (2.60%)	–
*HormTx compliance*		
Compliant	239 (28.3%)	–
Not compliant	294 (34.8%)	–
Unknown	312 (36.9%)	–

### Optimal quantile biomarkers in the screening cohort

The optimal quantiles for Ki-67 PCNA, PD-L2 and PR proteins with the corresponding median hazard ratio (HR*) in 100 split samples in the screening cohort are shown in Table 2. Table 2 presents also the optimism corrected hazard ratio (HR**) based on 200 bootstrap samples. The Additional file 1: Table S3 shows the three best (with the three highest effect sizes) quantile predictors identified in the screening data set.

For Ki-67, the 30th quantile had the highest median hazard ratio HR* (1.81) in split-sampling screening and the highest optimism corrected hazard ratio HR** (1.69; $95\%CI$:1.15$-$2.47). The 15th and 20th Ki-67 quantiles had slightly lower HR* and HR**, respectively (Table S3). None of the three best quantile predictors was significant in the multivariable Cox model adjusted for known clinico-pathologic risk factors (Table S3) because tumors with high 30th Ki-67 quantile had also higher histologic grade, larger tumor size and more likely to be Her2 positive (Table S3).

For PCNA, the 5th quantile had the highest median hazard ratio HR* (2.32) in split-sampling screening and the highest optimism corrected hazard ratio HR** (2.45; $95\%CI$:1.20$-$5.02). Meanwhile, the 10th and 15th PCNA quantiles had slightly lower HR* and HR**, respectively (Table S3). Similar to Ki-67, none of the three best PCNA quantile predictors was significant in the multivariable Cox model adjusted for known clinico-pathologic risk factors (Table S3).

For PD-L2, the 45th quantile had the highest median hazard ratio HR* (1.86) in split-sampling screening and the highest optimism corrected hazard ratio HR** (1.80; $95\% CI$: 1.25$-$2.57). The 50th and 55th PD-L2 quantiles had slightly lower HR* and HR**, respectively (Table S3). In contrast to cell proliferation markers Ki-67 and PCNA, PD-L2 quantile markers were significant in the multivariable Cox model adjusted for known clinico-pathologic risk factors (Table S5).

For PR, the 55th quantile had the highest effect size (HR*=0.44) in split-sampling screening and the highest optimism corrected effect size (HR**=0.47; $95\% CI$: 0.33$-$0.68). Meanwhile, the 25th and 30th PR quantiles had very similar HR* and HR** (Table S3). These quantile PR markers were also significant in the multivariable Cox model adjusted for known clinico-pathologic risk factors (Table S3).

**Table 2:** Optimal quantile predictors in the screening cohort with bootstrap-based optimism correction

Marker	Quantile	HR $^{*}$	Bootstrap adjusted univariable Cox				Bootstrap adjusted multivariable Cox			
			HR $$^{**}$$	95% Confidence Limits		p-value	HR $$^{**}$$	95% Confidence Limits		p-value
Ki-67	30	1.814	1.685	1.148	2.474	0.009	1.241	0.830	1.853	0.295
PCNA	5	2.324	2.452	1.197	5.022	0.016	1.614	0.775	3.361	0.203
PD-L2	45	1.860	1.792	1.250	2.567	0.002	1.854	1.281	2.685	0.001
PR	55	0.436	0.473	0.327	0.684	<0.001	0.637	0.430	0.945	0.027

* Median hazard ratio in 100 repeated split samples

** Optimism corrected hazard ratio using 200 bootstrap samples

### External validation results

The two types of cutoffs for dichotomization derived in the screening cohort (apparent performance cutoff and the median of the cutoffs for 100 random training set samples) were identical for Ki-67, PCNA, and PD-L2 and only slightly different for PR quantile biomarkers. Very small differences in the two types of cutoffs were observed for the second and third best quantile predictors. The corresponding results from the univariable and multivariable Cox models were very similar based on apparent performance cutoff and the median of the cutoffs for 100 random training set samples. Therefore, we report the external validation results based only on the apparent performance cutoffs derived in the screening cohort.

Table 3 presents the results for the dichotomized optimal quantile predictors, ccMSI, and percentages of Ki-67 positive cells in the univariable and multivariable Cox models fitted to the external validation cohort. Multivariable Cox models included the tumor size and lymph node positivity that were significant predictors of PFS before adding the biomarkers.

**Fig. 2:**
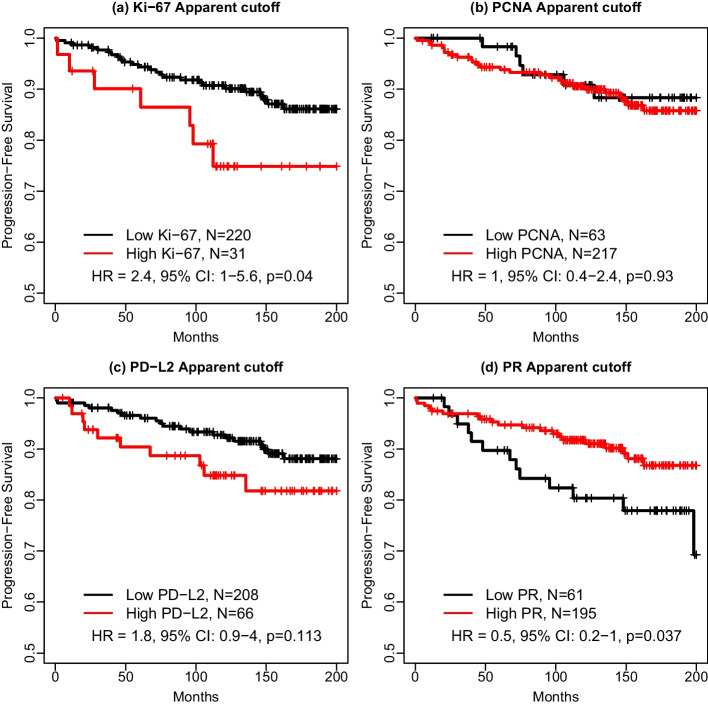
Kaplan-Meier estimators of the progression-free survival (PFS) by High vs. Low optimal quantile biomarkers in the external validation cohort **a**–**d** dichotomized Ki-67, PCNA, PD-L2, and PR biomarkers by apparent performance cutoff, respectively

In univariable analyses, the dichotomized Ki-67, PR and PD-L2, but not PCNA optimal quantile biomarkers were significant predictors of PFS. Only dichotomized PR ccMSI and PD-L2 ccMSI, but not Ki-67 ccMSI or PCNA ccMSI were significant predictors of PFS. Neither ccMSI nor quantile PCNA biomarkers were significant predictors of PFS. The Kaplan-Meier estimators of PFS by the optimal quantile predictors dichotomized using the apparent performance cutoff are shown in Fig. 2. For Ki-67 and PD-L2, the optimal quantile biomarkers have larger effect size as compared to dichotomized ccMSI biomarkers (HR=2.40 for Ki-67 30th percentile vs. 1.45 for Ki-67 ccMSI; HR=2.33 for PD-L2 45th percentile vs. 1.61 for PD-L2 ccMSI).

For PCNA, the effect size for the optimal quantile biomarker has somewhat larger than the effect size for ccMSI (HR=1.32 for PCNA 5th percentile vs. 1.22 for PCNA ccMSI) while for PR the effect size for ccMSI biomarker (HR=0.40) is slightly larger than the effect size for 55th percentile biomarker (HR=0.43).

Notable, the performance of the optimal quantile predictors is stable as the three best quantile predictors identified in the screening cohort yield similar results in the external validation cohort (Additional file 1: Table S4). Comparing the apparent performance cutoff in the entire screening data set with the median of the cutoffs for 100 random training set samples drawn from the screening data set, the two sets of cutoffs for majority of the three best quantile predictors are the same whereas the two types of cutoffs for the third best quantile predictor for Ki-67 (60th quantile) and the best quantile predictor for PR (55th quantile) are different. The median cutoff from 100 random split samples for Ki-67 yield larger effect size than the apparent performance cutoff (HR=2.38, $95\% CI$: 1.02$-$5.57, p=0.046) and the different cutoff for PR still produces the same effect size for PR.

The multivariable results were consistent with univariable results for all considered protein markers. The complete detail for the multivariable Cox models is provided in the Additional file 1: Table S5. Dichotomized PR ccMSI remained significant in the multivariable Cox model adjusting for grade and tumor size with smaller effect sized as compared to the optimal quantile biomarkers. The dichotomized PD-L2 and PR quantile biomarkers had significant HR in the univariable Cox model (HR for PD-L2 45th quantile: 2.33; $95\% CI$: 1.12$-$4.84; p=0.023; HR for PR 55th quantile: 0.43; $95\% CI$: 0.19$-$0.99; p=0.046, Table 3), and the effect was reduced and borderline significant in the multivariable Cox model (PD-L2 HR: 2.11; $95\% CI$: 0.98$-$4.56 0; p=0.057; PR HR: 0.45; $95\% CI$: 0.19$-$1.07; p=0.070; Table 3).

Since the number of events in the external validation cohort is limited, the data are not sufficient for a joint multivariable model with all considered proteins, but we have evaluated possible correlations among the optimal quantile biomarkers and did not observe any significant or substantial correlations (Additional file 1: Figure S1).

For Ki-67, none of the IKWG scoring at any cutoff among 5%, 15%, 20%, or 30% provided a significant hazard ratio of progression either in univariate models or multivariable models.

**Table 3:** Performance of the dichotomized optimal quantiles, ccMSIs, and percentages of Ki-67 positive cells in Cox model fitted to the external validation cohort

			Univariate Cox model				Multivariable Cox model			
Marker	Quantile	Cutoff$^{*}$	Optimal quantile				Optimal quantile			
			HR	95% Confidence Limits		p-value	HR	95% Confidence Limits		p-value
Ki-67	30	607.1	2.409	0.894	6.494	0.082	2.720	0.990	7.468	0.052
PCNA	5	1065.6	1.323	0.460	3.804	0.603	1.737	0.524	5.757	0.366
PD-L2	45	3938.5	2.331	1.121	4.846	0.023	2.110	0.977	4.557	0.057
PR	55	1052.6	0.431	0.188	0.985	0.046	0.447	0.187	1.068	0.070
		Cutoff$^{*}$	ccMSI				ccMSI			
			HR	95% Confidence Limits		p-value	HR	95% Confidence Limits		p-value
Ki-67		676.0	1.454	0.673	3.143	0.341	1.101	0.484	2.506	0.819
PCNA		8313.6	1.218	0.593	2.501	0.591	1.366	0.653	2.857	0.407
PD-L2		4309.2	1.607	0.798	3.234	0.184	1.375	0.664	2.846	0.391
PR		1119.0	0.402	0.179	0.903	0.027	0.406	0.171	0.961	0.040
		Cutoff$$^{**}$$	Ki-67 positive cells (%)				Ki-67 positive cells (%)			
			HR	95% Confidence Limits		p-value	HR	95% Confidence Limits		p-value
Ki-67		5	0.596	0.291	1.220	0.157	0.598	0.287	1.247	0.171
Ki-67		15	0.754	0.290	1.959	0.563	0.693	0.264	1.818	0.456
Ki-67		20	0.859	0.301	2.451	0.776	0.718	0.246	2.095	0.545
Ki-67		30	1.198	0.163	8.791	0.859	1.149	0.145	9.099	0.895

*Apparent cutoff based on the entire screening cohort without any bootstrap procedure

** Prespecified cutoffs for percentages of Ki-67 positive cells

## Discussion

Here, we propose an algorithm for simultaneously selecting and dichotomizing CSI distribution quantiles as optimal predictors of continuous, categorical or survival clinical outcome. The methods developed have been implemented in R (R Core Team, 2021) package Qindex, available at https://CRAN.R-project.org/package=Qindex. In the data analyzed, we had survival outcomes, but our package Qindex can be used with continuous or categorical (nominal or ordinal) outcomes with 3 or more categories.

The proposed approach involves two levels of model selection. The algorithm selects both the optimal CSI distribution quantile (among pre-specified candidates) and the optimal cutpoint for dichotomizing the optimal CSI distribution quantile. The selection of the optimal CSI cutpoint for dichotomization can be internally validated using the bootstrap-based optimism correction applied to a given quantile. In contrast, the selection of the optimal CSI quantile should be validated in an external data set.

For the proteins considered, our analysis resulted in externally validated optimal quantiles for cancer cell expression levels of Ki-67 (30th quantile), PD-L2 (45th quantile), and PR (55th quantile). The effect sizes in the external data set were comparable to the ones obtained in the screening cohort. The corresponding p-values were not significant at the standard level of 0.05 due to sample size limitation of the external cohort. For PCNA, none of the biomarkers derived in the screening cohort were significant predictors of PFS in the validation cohort. The discrepancy between the screening and validation cohorts may have contributed to differences in results for PCNA.

An optimal cutpoint for dichotomizing a continuous marker for predicting survival outcome may be also selected using the stand alone software package X-tile [31]. In contrast, our package makes use of R package rpart [23] to select the optimal dichotomization cutpoint. R package rpart is a more general tool as compared to X-tile because it can accommodate different types of outcomes and generate classification tree that goes well beyond selecting one or two cutpoints. For comparison, we performed optimal dichotomization of ccMSI markers using X-tile in our screening cohort as a training set. The resulting cutpoints were identical to the ones obtained using rpart. Respectively, our dichotomization approach using rpart and X-tile yield the same results for ccMSI markers in the independent validation set.

However, the main novelty and contribution of our work is to provide a tool for objective selection of the quantile(s) (of the marker expression distribution across all cancer cells) with the highest predictive value for the outcome of interest. In the data used in this study, the outcome of interest is progression-free survival, but our method and R package can be used also for categorical and continuous outcomes. Our additional contribution is the R function that computes bootstrap-based correction of optimistic bias for the effect size of a dichotomized continuous marker (see Additional file 2). Such adjusted effect size is not computed by X-tile.

Our work demonstrates that the quantiles computed for CSI distributions generated by histo-cytometry platforms or other single-cell technologies can be considered as predictors of progression-free survival in breast cancer. Since protein marker CSI distributions generated on other hardware-software platforms do not have to have the same scale as those presented here, it would be necessary to re-derive the corresponding optimal cutpoints for dichotomization. These optimal cutpoints can be internally validated using, for example, the bootstrap-based optimism correction procedure implemented in our R package Qindex.

This work was motivated by development of new cancer biomarkers based on distributions of protein expression level in tissues at the single-cell level. However, the proposed approach has broader utility for biomedical data with multiple measures of a candidate predictor per each subject. Other than histo-cytometry of protein markers, possible applications include *in situ* transcript profiling, flow cytometry and single cell RNAseq.

## Supplementary Information

**Additional file 1. Table S1:** Patient and tumor characteristics and association to dichotomized high or low Ki-67, PCNA, PD-L2, or PR levels in cancer cells for the screening cohort. **Table S2:** Patient and tumor characteristics and association to dichotomized high or low Ki-67, PCNA, PD-L2, or PR levels in cancer cells for the external validation cohort. **Table S3:** Three best quantile predictors identified in the screening data set. **Table S4:** Performance of the three best quantile predictors in the external validation cohort. **Table S5:** Performance of the optimal quantile biomarkers in the multivariable Cox model fitted to the external validation data. **Figure S1:** Scatter plots of log-transformed optimal quantile marker pairs. Subplot titles show the corresponding Spearman correlation coefficients (Rho) and p-values (p) for testing the null hypothesis that Rho=0. Only weak correlation was observed for Ki-67 log 30th quantile and PCNA log 5th quantile (a) and for PD-L2 log 45th quantile and PCNA log 5th quantile (d). All other pairs of optimal quantiles are not correlated (b, c, e, f).

**Additional file 2.** This file contains the R source code.

## Data Availability

The R code and part of the data used for analyses are publicly available online as part of the CRAN package Qindex https://CRAN.R-project.org/package=Qindex. The rest of the data can be obtained from the corresponding authors upon request.

## Abbreviations

CBCP: Clinical Breast Care Program
CI: Confidence Interval
CSI: Cell Signal Intensity
ER: Estrogen Receptor
HR: Hazard Ratio
IHC: Immunohistorychemistry
IKWG: International Ki67 in ﻿Breast Cancer Working Group
MICE: Multivariable Imputation by Chained Equations
ccMSI: cancer compartment Mean Signal Intensity
OR: Odds Ratio
PCNA: Proliferating Cell Nuclear Antigen
PD-1: Programmed Cell Death Protein 1
PD-L2: Programmed cell Death 1 ligand 2
PFS: Progression-free Survival
PR: Progesterone Receptor
TMA: Microarray
