# Supplementary material for: Selection of optimal quantile protein biomarkers based on cell-level immunohistochemistry data
Source: BMC Bioinformatics. 2023 Jul 22;24:298. doi: 10.1186/s12859-023-05408-8 (PMC10363294; doi:10.1186/s12859-023-05408-8)
Supplement: Supplementary file 1 — Additional file 1. Table S1: Patient and tumor characteristics and association to dichotomized high or low Ki-67, PCNA, PD-L2, or PR levels in cancer cells for the screening cohort. Table S2: Patient and tumor characteristics and association to dichotomized high or low Ki-67, PCNA, PD-L2, or PR levels in cancer cells for the external validation cohort. Table S3: Three best quantile predictors identified in the screening data set. Table S4: Performance of the three best quantile predictors in the external validation cohort. Table S5: Performance of the optimal quantile biomarkers in the multivariable Cox model fitted to the external validation data. Figure S1: Scatter plots of log-transformed optimal quantile marker pairs. Subplot titles show the corresponding Spearman correlation coefficients (Rho) and p-values (p) for testing the null hypothesis that Rho=0. Only weak correlation was observed for Ki-67 log 30th quantile and PCNA log 5th quantile (a) and for PD-L2 log 45th quantile and PCNA log 5th quantile (d). All other pairs of optimal quantiles are not correlated (b, c, e, f). [file 12859_2023_5408_MOESM1_ESM.pdf]

Additional File 1

Table S1: Patient and tumor characteristics and association to dichotomized high or low Ki-67, PCNA, PD-L2, or PR levels in cancer cells for the screening cohort

Table S2: Patient and tumor characteristics and association to dichotomized high or low Ki-67, PCNA, PD-L2, or PR levels in cancer cells for the external validation cohort

Table S3: Three best quantile predictors identified in the screening data set

Table S4: Performance of the three best quantile predictors in the external validation cohort

Table S5: Performance of the optimal quantile biomarkers in the multivariable Cox model fitted to the external validation data

Figure S1: Scatter plots of log-transformed optimal quantile marker pairs. Subplot titles show the corresponding Spearman correlation coefficients (Rho) and p-values (p) for testing the null hypothesis that Rho=0. Only weak correlation was observed for Ki-67 log 30th quantile and PCNA log 5th quantile (a) and for PD-L2 log 45th quantile and PCNA log 5th quantile (d). All other pairs of optimal quantiles are not correlated (b, c, e, f).

Table S1: Patient and tumor characteristics and association to dichotomized high or low Ki-67, PCNA, PD-L2, or PR levels in cancer cells (screening cohort)

Marker	Ki-67			PCNA			PD-L2			PR		
	High N=136	Low N=486	p-value	High N=588	Low N=112	p-value	High N=219	Low N=464	p-value	High N=467	Low N=163	p-value
Recurrence:			0.002			0.002			0.001			<0.001
Recurred	39 (28.7%)	79 (16.3%)		116 (19.7%)	8 (7.14%)		55 (25.1%)	65 (14.0%)		68 (14.6%)	49 (30.1%)	
Not Recurred	97 (71.3%)	407 (83.7%)		472 (80.3%)	104 (92.9%)		164 (74.9%)	399 (86.0%)		399 (85.4%)	114 (69.9%)	
Age	58.0 (14.5)	60.5 (12.6)	0.065	59.4 (13.6)	62.9 (11.3)	0.004	60.1 (13.7)	59.4 (13.0)	0.521	59.4 (13.4)	60.5 (12.3)	0.350
Race:			0.845			0.006			0.180			0.273
White	116 (85.3%)	420 (86.4%)		495 (84.2%)	106 (94.6%)		181 (82.6%)	403 (86.9%)		405 (86.7%)	135 (82.8%)	
Non-white	20 (14.7%)	66 (13.6%)		93 (15.8%)	6 (5.36%)		38 (17.4%)	61 (13.1%)		62 (13.3%)	28 (17.2%)	
Histological Grade:			0.004			<0.001			0.110			<0.001
1	36 (26.5%)	183 (37.7%)		189 (32.1%)	48 (42.9%)		65 (29.7%)	171 (36.9%)		177 (37.9%)	32 (19.6%)	
2	54 (39.7%)	205 (42.2%)		251 (42.7%)	53 (47.3%)		96 (43.8%)	201 (43.3%)		194 (41.5%)	76 (46.6%)	
3/4	45 (33.1%)	96 (19.8%)		146 (24.8%)	10 (8.93%)		56 (25.6%)	90 (19.4%)		94 (20.1%)	53 (32.5%)	
Missing	1 (0.74%)	2 (0.41%)		2 (0.34%)	1 (0.89%)		2 (0.91%)	2 (0.43%)		2 (0.43%)	2 (1.23%)	
Tumor size:			0.095			0.003			0.559			<0.001
Tumor Size < 2 cm	74 (54.4%)	314 (64.6%)		358 (60.9%)	85 (75.9%)		132 (60.3%)	299 (64.4%)		316 (67.7%)	79 (48.5%)	
Tumor Size 2-5 cm	48 (35.3%)	133 (27.4%)		168 (28.6%)	24 (21.4%)		66 (30.1%)	123 (26.5%)		125 (26.8%)	59 (36.2%)	
Tumor Size > 5 cm	14 (10.3%)	39 (8.02%)		62 (10.5%)	3 (2.68%)		21 (9.59%)	42 (9.05%)		26 (5.57%)	25 (15.3%)	
Node:			0.754			0.045			1.000			0.146
Positive	51 (37.5%)	171 (35.2%)		217 (36.9%)	29 (25.9%)		81 (37.0%)	171 (36.9%)		152 (32.5%)	65 (39.9%)	
Negative	85 (62.5%)	314 (64.6%)		368 (62.6%)	82 (73.2%)		137 (62.6%)	291 (62.7%)		313 (67.0%)	97 (59.5%)	
Unknown	0 (0.00%)	1 (0.21%)		3 (0.51%)	1 (0.89%)		1 (0.46%)	2 (0.43%)		2 (0.43%)	1 (0.61%)	
Her2:			0.066			0.533			0.970			0.020
Positive	21 (15.4%)	42 (8.64%)		60 (10.2%)	8 (7.14%)		21 (9.59%)	43 (9.27%)		40 (8.57%)	27 (16.6%)	
Negative	109 (80.1%)	423 (87.0%)		463 (78.7%)	93 (83.0%)		177 (80.8%)	374 (80.6%)		412 (88.2%)	132 (81.0%)	
Missing	6 (4.41%)	21 (4.32%)		65 (11.1%)	11 (9.82%)		21 (9.59%)	47 (10.1%)		15 (3.21%)	4 (2.45%)	
Chemotherapy:			0.048			0.040			0.301			0.852
Chemotherapy: Yes	44 (32.4%)	117 (24.1%)		169 (28.7%)	21 (18.8%)		65 (29.7%)	132 (28.4%)		123 (26.3%)	46 (28.2%)	
Chemotherapy: No	91 (66.9%)	351 (72.2%)		405 (68.9%)	86 (76.8%)		151 (68.9%)	316 (68.1%)		331 (70.9%)	112 (68.7%)	
Unknown	1 (0.74%)	18 (3.70%)		14 (2.38%)	5 (4.46%)		3 (1.37%)	16 (3.45%)		13 (2.78%)	5 (3.07%)	
Radiation:			0.241			0.018			0.070			0.670
Radiation: Yes	52 (38.2%)	189 (38.9%)		218 (37.1%)	53 (47.3%)		77 (35.2%)	185 (39.9%)		176 (37.7%)	67 (41.1%)	
Radiation: No	83 (61.0%)	280 (57.6%)		358 (60.9%)	54 (48.2%)		140 (63.9%)	264 (56.9%)		279 (59.7%)	91 (55.8%)	
Unknown	1 (0.74%)	17 (3.50%)		12 (2.04%)	5 (4.46%)		2 (0.91%)	15 (3.23%)		12 (2.57%)	5 (3.07%)	
HormTx compliance:			0.465			0.512			0.486			0.010
Compliant	37 (27.2%)	134 (27.6%)		168 (28.6%)	35 (31.2%)		70 (32.0%)	129 (27.8%)		127 (27.2%)	43 (26.4%)	
Not compliant	57 (41.9%)	178 (36.6%)		201 (34.2%)	32 (28.6%)		74 (33.8%)	159 (34.3%)		161 (34.5%)	76 (46.6%)	
Unknown	42 (30.9%)	174 (35.8%)		219 (37.2%)	45 (40.2%)		75 (34.2%)	176 (37.9%)		179 (38.3%)	44 (27.0%)	

Table S2: Patient and tumor characteristics and association to dichotomized high or low Ki-67, PCNA, PD-L2, or PR levels in cancer cells (external validation cohort)

Marker	Ki-67			PCNA			PD-L2			PR		
	High N=32	Low N=219	p-value	High N=225	Low N=55	p-value	High N=69	Low N=205	p-value	High N=196	Low N=60	p-value
Recurrence:			0.150			0.399			0.105			0.036
Recurred	7 (21.9%)	25 (11.4%)		28 (12.4%)	4 (7.27%)		12 (17.4%)	19 (9.27%)		20 (10.2%)	13 (21.7%)	
Not Recurred	25 (78.1%)	194 (88.6%)		197 (87.6%)	51 (92.7%)		57 (82.6%)	186 (90.7%)		176 (89.8%)	47 (78.3%)	
Age	55.3 (13.5)	60.2 (13.3)	0.063	59.0 (13.4)	60.9 (12.6)	0.320	56.2 (12.8)	60.2 (13.3)	0.031	59.2 (13.7)	62.1 (13.4)	0.145
Race:			1.000			0.482			0.267			0.445
White	25 (78.1%)	170 (77.6%)		176 (78.2%)	46 (83.6%)		52 (75.4%)	169 (82.4%)		152 (77.6%)	43 (71.7%)	
Non-white	7 (21.9%)	49 (22.4%)		49 (21.8%)	9 (16.4%)		17 (24.6%)	36 (17.6%)		44 (22.4%)	17 (28.3%)	
Histological Grade:			<0.001			0.147			0.017			0.018
1	4 (12.5%)	95 (43.4%)		81 (36.0%)	27 (49.1%)		19 (27.5%)	87 (42.4%)		83 (42.3%)	18 (30.0%)	
2	15 (46.9%)	89 (40.6%)		99 (44.0%)	23 (41.8%)		29 (42.0%)	86 (42.0%)		84 (42.9%)	23 (38.3%)	
3/4	13 (40.6%)	34 (15.5%)		44 (19.6%)	5 (9.09%)		21 (30.4%)	31 (15.1%)		28 (14.3%)	19 (31.7%)	
Missing	0 (0.00%)	1 (0.46%)		1 (0.44%)	0 (0.00%)		0 (0.00%)	1 (0.49%)		1 (0.51%)	0 (0.00%)	
Tumor size:			0.037			0.435			<0.001			0.106
Tumor Size < 2 cm	13 (40.6%)	139 (63.5%)		137 (60.9%)	39 (70.9%)		27 (39.1%)	141 (68.8%)		111 (56.6%)	43 (71.7%)	
Tumor Size 2-5 cm	16 (50.0%)	66 (30.1%)		74 (32.9%)	14 (25.5%)		35 (50.7%)	53 (25.9%)		72 (36.7%)	14 (23.3%)	
Tumor Size > 5 cm	3 (9.38%)	14 (6.39%)		14 (6.22%)	2 (3.64%)		7 (10.1%)	11 (5.37%)		13 (6.63%)	3 (5.00%)	
Node:			0.940			0.884			0.576			0.111
Positive	12 (37.5%)	76 (34.7%)		75 (33.3%)	19 (34.5%)		25 (36.2%)	65 (31.7%)		69 (35.2%)	22 (36.7%)	
Negative	19 (59.4%)	134 (61.2%)		143 (63.6%)	34 (61.8%)		43 (62.3%)	131 (63.9%)		122 (62.2%)	33 (55.0%)	
Unknown	1 (3.12%)	9 (4.11%)		7 (3.11%)	2 (3.64%)		1 (1.45%)	9 (4.39%)		5 (2.55%)	5 (8.33%)	
Her2:			0.182			0.667			0.502			0.127
Positive	5 (15.6%)	13 (5.94%)		20 (8.89%)	3 (5.45%)		8 (11.6%)	16 (7.80%)		11 (5.61%)	8 (13.3%)	
Negative	27 (84.4%)	205 (93.6%)		204 (90.7%)	52 (94.5%)		61 (88.4%)	188 (91.7%)		184 (93.9%)	52 (86.7%)	
Missing	0 (0.00%)	1 (0.46%)		1 (0.44%)	0 (0.00%)		0 (0.00%)	1 (0.49%)		1 (0.51%)	0 (0.00%)	

Table S3: Three best quantile predictors identified in the screening data set

Marker	Quantile	HR *	Bootstrap adjusted univariable Cox				Bootstrap adjusted multivariable Cox			
			HR **	HR 95% CI		p-value	HR **	HR 95% CI		p-value
Ki-67	30	1.814	1.685	1.148	2.474	0.009	1.241	0.830	1.853	0.295
	20	1.649	1.638	1.116	2.404	0.013	1.192	0.799	1.781	0.392
	60	1.654	1.630	1.130	2.351	0.010	1.056	0.717	1.554	0.784
PCNA	5	2.324	2.452	1.197	5.022	0.016	1.614	0.775	3.361	0.203
	10	2.158	2.352	1.192	4.639	0.015	1.504	0.749	3.019	0.254
	15	2.070	2.253	1.142	4.443	0.021	1.545	0.769	3.102	0.224
PD-L2	45	1.860	1.792	1.250	2.567	0.002	1.854	1.281	2.685	0.001
	55	1.847	1.823	1.272	2.610	0.001	1.881	1.299	2.723	0.001
	50	1.837	1.792	1.250	2.567	0.002	1.854	1.281	2.685	0.001
PR	55	0.436	0.473	0.327	0.684	$<$0.001	0.637	0.430	0.945	0.027
	30	0.449	0.467	0.323	0.676	$<$0.001	0.614	0.412	0.914	0.018
	25	0.450	0.475	0.329	0.687	$<$0.001	0.603	0.407	0.894	0.013

* Median hazard ratio in 100 repeated split samples
** Optimism corrected hazard ratio using 200 bootstrap samples

Table S4: Performance of the three best quantile predictors in the external validation cohort

Marker	Quantile	Cutoff *	Optimal quantile			
			HR **	HR 95% CI		p-value
Ki-67	30	607.1 ***	2.409	0.894	6.494	0.082
	20	562.2 ***	2.409	0.894	6.494	0.082
	60	707.9	1.336	0.498	3.581	0.565
PCNA	5	1065.6	1.323	0.460	3.804	0.603
	10	1221.2	1.542	0.537	4.430	0.421
	15	1346.6	1.140	0.435	2.987	0.790
PD-L2	45	3938.5	2.331	1.121	4.846	0.023
	55	4542.8	2.170	1.031	4.567	0.041
	50	4158.2	2.281	1.097	4.743	0.027
PR	55	1052.6	0.431	0.188	0.985	0.046
	30	847.0	0.479	0.205	1.121	0.090
	25	827.4	0.483	0.206	1.130	0.093

* Apparent cutoff based on the entire screening cohort without any bootstrap procedure
** Hazard ratio based on optimal quantile
*** Note that 20th quantile and 30th quantile results are the same because of fairly close cutoffs

Table S5: Performance of the optimal quantile biomarkers in the multivariable Cox model fitted to the external validation data

Marker	Variable	HR	HR 95% CI		p-value
Ki-67	30th quantile High vs. Low	2.720	0.990	7.468	0.052
	Node positive vs. negative	2.287	0.926	5.651	0.073
	TstageTumor 2-5 vs. <2	0.984	0.366	2.650	0.975
	TstageTumor >5 vs. <2	4.162	1.389	12.471	0.011
PCNA	5th quantile High vs. Low	1.737	0.524	5.757	0.366
	Node positive vs. negative	1.963	0.897	4.296	0.091
	TstageTumor 2-5 vs. <2	1.521	0.655	3.533	0.329
	TstageTumor >5 vs. <2	3.616	1.189	10.999	0.024
PD-L2	45th quantile High vs. Low	2.110	0.977	4.557	0.058
	Node positive vs. negative	1.896	0.875	4.107	0.105
	TstageTumor 2-5 vs. <2	1.349	0.570	3.196	0.496
	TstageTumor >5 vs. <2	3.708	1.265	10.874	0.017
PR	55th quantile High vs. Low	0.447	0.188	1.068	0.070
	Node positive vs. negative	2.236	0.882	5.669	0.090
	TstageTumor 2-5 vs. <2	1.736	0.650	4.633	0.271
	TstageTumor >5 vs. <2	5.345	1.698	16.822	0.004

Figure S1: Scatter plots of log-transformed optimal quantile marker pairs

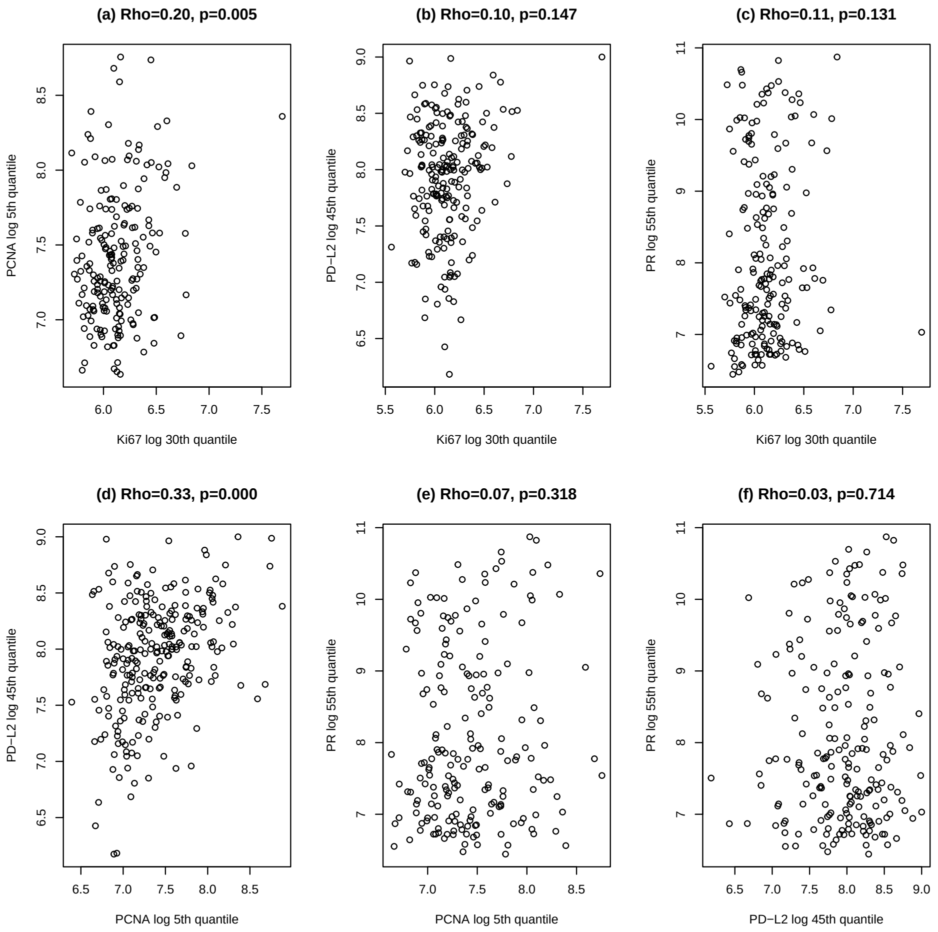

Subplot titles show the corresponding Spearman correlation coefficients (Rho) and p-values (p) for testing the null hypothesis that Rho=0. Only weak correlation was observed for Ki-67 log 30th quantile and PCNA log 5th quantile (a) and for PD-L2 log 45th quantile and PCNA log 5th quantile (d). All other pairs of optimal quantiles are not correlated (b, c, e, f).
