# Supplementary material for: Selection of optimal quantile protein biomarkers based on cell-level immunohistochemistry data
Source: BMC Bioinformatics. 2023 Jul 22;24:298. doi: 10.1186/s12859-023-05408-8 (PMC10363294; doi:10.1186/s12859-023-05408-8)
Supplement: Supplementary file 2 — Additional file 2. This file contains the R source code. [file 12859_2023_5408_MOESM2_ESM.pdf]

```
# Example of using 'Qindex' with test data is available in help files
(see ?`Qindex-package`)
#
# All data sets include the following variables:
#
#  "PATIENT_ID" -- Unique Patient ID
#  "tissueID" -- ID of the TMA core
#  "RECURRENCE" -- Recurrence indicator, 1=Recurred, 0=not Recurred
#  "RECFREESURV_MO" -- Recurrence-free Survival Time in months
#  "Marker" -- Cell Signal Intensity of the protein immunofloerscence signal
#  "inner_x" x-coordinate in the cell centroid in the TMA core
#  "inner_y" y-coordinate in the cell centroid in the TMA core
#  "AGE_AT_DX" -- Age at Diagnosis
#  "Tstage" -- Tumor stage
#  "NodeSt" -- Node stage, -1=unknown, 0=Node Negative, 1=Node Positive
#  "HRpos" -- Indicator of Hormone Positive status (ER+ or PR+), 1 = positive
#   "HistologicalGrade" -- Histology grade
#   "Her2_path_qIF" -- Her2 status, 1 = positive, 0 = negative
#   "RACE" -- Race, White, Black, Asian, Native Hawaiian or Other Pacific Islander,
American Indian or Alaska Native, Unknown
#   "RadjCHEMO" -- Adjuvant Chemo Tx category, 0 = unknown,  1 = done, 2 = NOT done
#   "RadjRAD" -- Adjuvant Radiation Tx category, 0 = unknown,  1 = done, 2 =
NOTdone
#   "HORM_4cat" -- Hormone Tx category, 0=unknown, 1 = not indicated, 2 = done, 3 =
recommended, but not done
#   "MSI" -- Mean Signal Intensity (mean over all cells in the TMA core)}

#
# In this supplementary material, we show the analysis of `Ki67`

require('Qindex')
library(Qindex)

##########################################################################################
# Computation of quantiles and selection of optimal quantile as a predictor of
survival outcome
##########################################################################################

# I. Function clusterQp calculates quantiles for variable Marker in input data=Ki67
Ki67_Qps = clusterQp(data = Ki67, subjID = 'PATIENT_ID',
  exclude = c('tissueID','inner_x','inner_y'), contX = 'Marker')
head(Ki67_Qps)
sapply(Ki67_Qps, FUN = class)
head(Ki67_Qps$Marker)

# The object train_Qps returned by 'clusterQp' includes a matrix variable 'Marker'

# II. Function optimal_splitSample_dichotom selects optimal quantile biomarkers
# set.seed if needed
Ki67c = optim_splitSample_dichotom(Surv(RECFREESURV_MO, RECURRENCE) ~ Marker, data
= Ki67_Qps,
  nsplit = 20L, top = 2L) # slow
head(Ki67c)
```

```
attr(Ki67c, 'top') # under-the-hood statistics for the optimal predictors

# III. Function 'BBC_dichotom' computes optimism corrected hazard ratio for optimal
quantiles
# set.seed here if needed
mod_c = BBC_dichotom(Surv(RECFREESURV_MO, RECURRENCE) ~ 1,
  data = Ki67c, contX = 'Marker', R = 100L)
summary(mod_c)

# The object mod_c includes bootstrap-based dichotomizaton thresholds
attr(mod_c, 'median_optimism')
attr(attr(mod_c, 'median_optimism'), 'boot_branch')
attr(mod_c, 'apparent_branch')
```
